# An EMG-based feature extraction method using a normalized weight vertical visibility algorithm for myopathy and neuropathy detection

**DOI:** 10.1186/s40064-016-3772-2

**Published:** 2016-12-20

**Authors:** Patcharin Artameeyanant, Sivarit Sultornsanee, Kosin Chamnongthai

**Affiliations:** 1 Department of Electronic and Telecommunication Engineering, Faculty of Engineering, King Mongkut’s University of Technology Thonburi, 126 Pracha-uthit Rd., Bangmod, Thungkhru, Bangkok, 10140 Thailand; 2 School of Business, University of the Thai Chamber of Commerce, 126/1 Vibhavadi Rd., Dindang, Bangkok, 10400 Thailand

**Keywords:** EMG signal, Complex network, Normalized weight vertical visibility algorithm, Network measurements, *k*-Nearest neighbor, Multilayer perceptron neural network, Support vector machine

## Abstract

**Background:**

Electromyography (EMG) signals recorded from healthy, myopathic, and amyotrophic lateral sclerosis (ALS) subjects are nonlinear, non-stationary, and similar in the time domain and the frequency domain. Therefore, it is difficult to classify these various statuses.

**Methods:**

This study proposes an EMG-based feature extraction method based on a normalized weight vertical visibility algorithm (NWVVA) for myopathy and ALS detection. In this method, sampling points or nodes based on sampling theory are extracted, and features are derived based on relations among the vertical visibility nodes with their amplitude differences as weights. The features are calculated via selective statistical mechanics and measurements, and the obtained features are assembled into a feature matrix as classifier input. Finally, powerful classifiers, such as *k*-nearest neighbor, multilayer perceptron neural network, and support vector machine classifiers, are utilized to differentiate signals of healthy, myopathy, and ALS cases.

**Results:**

Performance evaluation experiments are carried out, and the results revealed 98.36% accuracy, which corresponds to approximately a 2% improvement compared with conventional methods.

**Conclusions:**

An EMG-based feature extraction method using a NWVVA is proposed and implemented to detect healthy, ALS, and myopathy statuses.

## Background

Currently, amyotrophic lateral sclerosis (ALS), or neuropathy, a rapidly progressive, invariably fatal neurological disease that affects the neurons responsible for controlling voluntary muscles in the arms, legs, and face (Ahdab et al. 2013), is diagnosed in approximately 6000 people each year (ALS Association 2016). In the USA alone, the number of patients is estimated to be as many as 20,000. This disease belongs to a group of motor neuron disorders and eventually leads to death. According to previous studies, patients who are diagnosed live an average of 3 years, and 20, 10, and 5% of them die in 5, 10, and 20 years, respectively. Myopathy is a neuromuscular disorder that causes muscle cramps, stiffness, and spasms, and muscle weakness is the primary symptom due to dysfunction of muscle fibers and eventually causes death. In accordance with the 2005 statistics data of the USA (Oskarsson 2011), approximately 2.97 million patients have been diagnosed with myopathy. In diagnosing both aforementioned diseases, medical doctors first interview patients, although sometimes the patients are extremely weak and unavailable to even speak. In such cases, electromyography (EMG) is used to analyze muscle signals to assist a specialized neurological expert to diagnose both myopathy and ALS (Kincaid 2015; Weiss et al. 2015; Gitiaux et al. 2016). However, the number of neurological experts is quite limited, and therefore, an automatic system to assist diagnosis is urgently required. Such a system could be used not only for assisting diagnosis but also for periodic detection and monitoring. In performing diagnoses based on EMG signals, a primary issue is that the system must correctly classify an EMG signal as ALS or myopathic, because different therapies and drugs are used to treat the two disorders.

In studying and developing this kind of system, EMG signals is regarded as an excellent approach for acquiring data (Yousefi and Hamilton-Wright 2014), which records the corresponding electrical to activity of motor units in the neuromuscular system. Analysis of EMG signals is generally performed in two cases. The first is for prosthetic device control and human–machine interactions (Naik and Kumar 2011; Naik et al. 2014, 2016a; Arjunan et al. 2014, 2015; Guo et al. 2015; Naik and Nguyen 2015). The second is for diagnosing disorders (Xie et al. 2014). Neuromuscular disorders are related to pathological changes in the structure of the motor unit and can be generally divided into two categories: muscular (myopathy) and neuronal (neuropathy) (Nikolic 2001) disorders. The need for distinct classification between myopathy and neuropathy originates from the differences between the causes of the diseases, which is a critical factor in determining treatment. The development of a highly accurate diagnostic system based on EMG readings would provide a promising way to improve the assessment of neuromuscular disorders (Gokgoz and Subasi 2015). Highly accurate classification problems depend on the crucial step of feature extraction. If features are extracted sufficiently well, it is possible to obtain outstanding classification performance.

Previous studies related to feature extraction of EMG signals have been proposed in three main domains, the frequency domain, the time–frequency domain, and the complex network domain. In frequency analyses, fast Fourier transform (FFT) and autoregressive (AR) spectral models have been employed to extract features (Guler and Kocer 2005; Subasi et al. 2006; Kocer 2010; Sultornsanee et al. 2011). Power spectral analysis of FFT and AR can represent the characteristics of the signal. However, different subjects have different signal strengths in addition to nonlinearity and chaos. Various types of wavelets have been used to analyze EMG signals in the time–frequency domain (Gokgoz and Subasi 2015; Hu et al. 2005; Istenic et al. 2010; Subasi 2012a, b, 2013a, b). The advantage of the method is the ability to perform analyses in various sub-bands. However, computational complexity might occur at the initial stages, such as when selecting the mother wavelet. Additionally, the level of decomposition is related to the number of sub-bands. Using many sub-bands with various features in each sub-band results in a high dimension of input for the classifier. Mishra et al. (2016) and Naik et al. (2016b) utilized an empirical mode decomposition technique to analyze EMG signals, which was proven to be quite versatile over a broad range of applications for extracting signals from data generated in noisy nonlinear and non-stationary processes.

Finally, for the complex network domain, Campanharo et al. (2011) studied the duality between the time series and networks and proposed a map of the time series resulting in networks with distinct topological properties. Thus, nonlinear signals can be transformed into a complex network using a visibility algorithm. Lacasa et al. (2007) proposed a visibility algorithm to convert a time series signal into a graph. The resulting graph inherited several properties of the series in its structure. Luque et al. (2009) employed a horizontal visibility algorithm, which is a geometrically simpler and an analytically solvable version of the visibility algorithm. All the aforementioned works on the complex network domain are pure theoretical concepts without evidence of implementation in signal analysis. Tang et al. (2013) used visibility graphs from higher frequency bands to classify electroencephalogram (EEG) signals. They concluded that their approach is better than the simple entropy method. Additionally, Zhu et al. (2012) employed visibility graphs with nonlinear feature extraction algorithms on the EEG signal, although their algorithms were slower than FFT analysis, which is not suitable for practical purposes. Subsequently, Zhu et al. (2014) introduced the fast-weighted horizontal visibility algorithm (FHVA). The FHVA can be employed using signals that have high amplitude variations. However, the FHVA is not suitable for EMG signals because the algorithm uses a horizontal relationship, which does not distinguish features sufficiently well; thus, the classification results using this method are incorrect.

In our previous works, Artameyanant et al. (2014) proposed a feature extraction technique based on transforming the signal into a complex network using a vertical visibility algorithm. The method yielded excellent accuracy results. However, a rapidly decreasing/increasing signal configuration could yield the same features. Therefore, a classification error could occur. The authors then improved upon the work in Artameyanant et al. (2014) by presenting a weight-visibility algorithm for transforming the signal into a complex network (Artameyanant et al. 2015). The method solved the problem of the same features being yielded for a different type of signal. However, the drawback was the loss of the link in the calculation caused by the same amplitude of the signal. Additionally, the EMG signal of each subject for the same type of disease can vary in signal strength. Thus, the various strengths of signals for different patients can induce classification problems. In this paper, we overcome the drawbacks of our previous work with two steps of feature extraction. First, we propose normalizing the signal with respect to the maximum/minimum value of each epoch. The normalized signal corresponds to the visual inspection of the same scale of the signal pattern by neurological expertise for classification. Second, we introduce an adjusted-weight vertical visibility algorithm to obtain the adjacency matrix for network measurements. The proposed work shows that feature extraction based on network measurements of the adjusted-weight vertical visibility algorithm can be used as an analysis tool for EMG signals. Some distinct characteristics inherited in the signal are extracted and employed as a feature vector. Performance is evaluated using several types of classifiers: *k*-NN, MLPNN, and SVM. The proposed method yields outstanding average accuracy results.

The organization of the paper is as follows. In “EMG signal analysis and basic concept” section, we analyze the research problem and outline the basic concept. In “Proposed method of EMG-based feature extraction” section, we explain the proposed method according to the basic concept. We describe the datasets and experimental results in “Datasets and experimental results” section, and discuss errors and trade-offs in “Discussion” section. Finally, the research is concluded in “Conclusions” section.

## EMG signal analysis and basic concept

To select efficient tools for feature extraction and classification, we analyze the EMG signal and explain our ideas in this section.

**Fig. 1:**
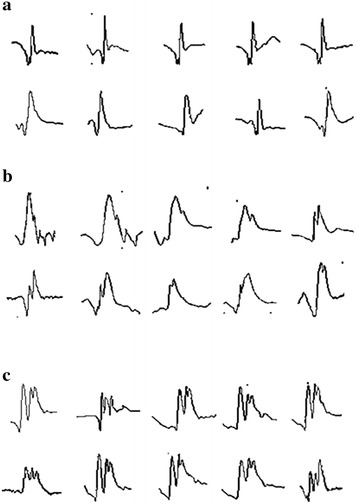
Samples of EMG signals in normal, ALS, and myopathy groups. **a** Normal, **b** ALS, and **c** myopathy

**Fig. 2:**
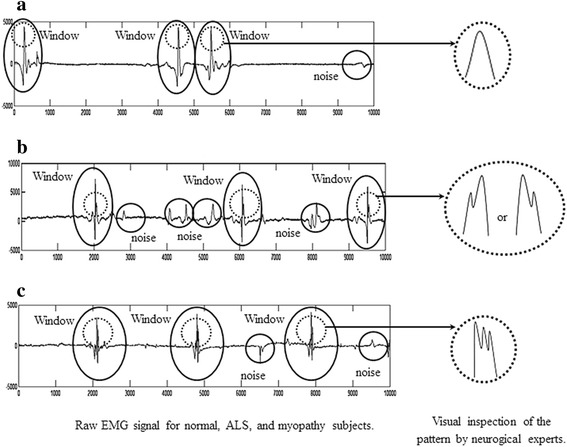
Characteristics of EMG signals. **a** Normal, **b** ALS, and **c** myopathy

The EMG signal originally has a non-periodic and non-stationary character. As shown by some samples in Fig. 1, the EMG signals in the normal (healthy), ALS, and myopathy groups, which are exactly not the same in each group, seem to have its own pattern. These signals apparently can be identified as one of three types, normal (healthy), ALS, or myopathic, by neurological experts who are practically trained to specifically identify ALS. For instance, as shown by the part of the signal surrounded by ellipses in the first column, which are expanded in the second column in Fig. 2, normal, ALS, and myopathic signals have different apparent features: a peak of 400 μV with an average pulse duration of 15 ms, a peak of 1400 μV with a duration of 20 ms, and a peak of 300 μV with duration of 10 ms (Nikolic 2001), respectively. In fact, these time-domain signal features are therefore considered as specific patterns for neurological experts to inspect as ALS and myopathic statuses. Analytically, an EMG signal is composed of muscle responses and noise, as indicated by ellipses and circles, respectively, in Fig. 2. Muscle responses are the target, which need to be separated from the other signal components. This can be done by determining a threshold value in advance for classifying peaks of muscle responses, which are normally located in the upper layer, and then opening windows of muscle responses for classification. In the window (ellipse), the peak parts shown by the dash-line circles in Fig. 2 are highlighted and considered as normal, ALS, and myopathic patterns, and the results are illustrated in the second column of Fig. 2. In conclusion, the detection of one, two, or three detected peaks represent normal, ALS, and myopathic, respectively, as shown in Fig. 2.

**Fig. 3:**
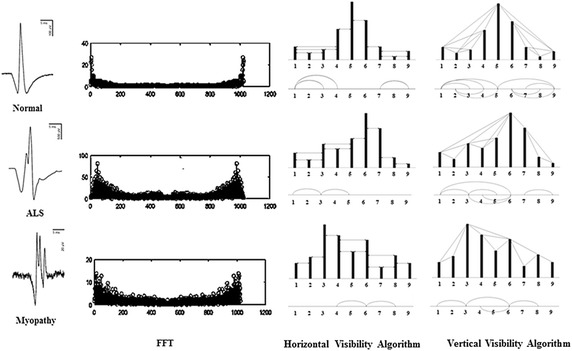
Comparison between the horizontal visibility algorithm and vertical visibility algorithm for the normal, ALS, and myopathic signals

**Fig. 4:**
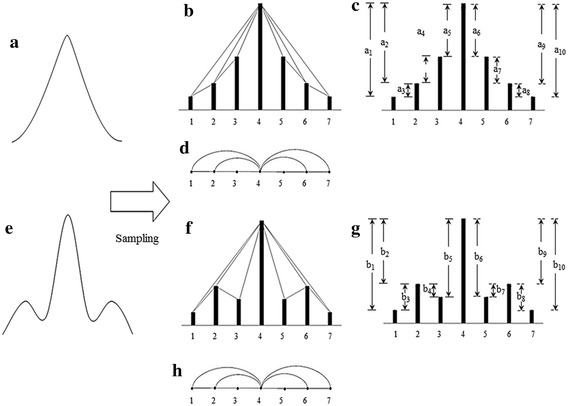
An example of different epochs yielding the same number of nodes and links. **a** Epoch A, **b** sampling pulse of epoch A, **c** a_i_ = a_1_, a_2_, a_3_,… a_n_, **d** vertical visibility algorithm of epoch B, **e** epoch B, **f** sampling pulse of epoch B, **g** b_i_ = b_1_, b_2_, b_3_,… b_n_, and **h** vertical visibility algorithm of epoch B

**Fig. 5:**
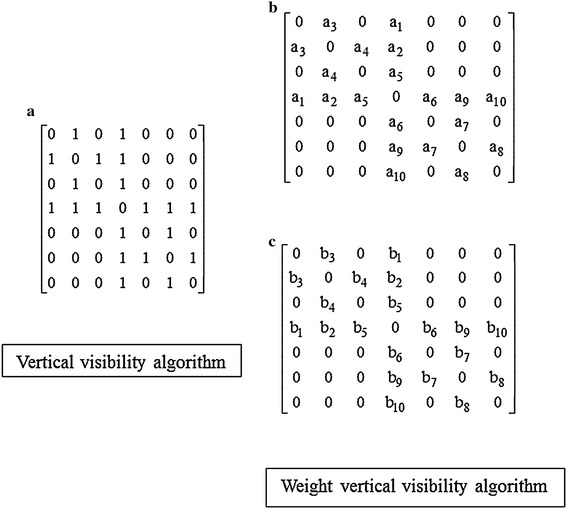
Matrices representing the number of links (**a**) and weights (**b**, **c**)

In analyzing the specific apparent patterns of normal, ALS, and myopathy signal parts shown in the first column of Fig. 3, the power spectrum density of the three signals transformed by FFT are nearly the same, as shown by the samples in the second column of Fig. 3. This indicates that it is likely impossible to differentiate these signals, especially myopathy and ALS statuses, by such transformation of signals to the frequency domain. Neurological experts generally observe signals in the time domain, extract epochs of specific parts of the signal, and classify the signals by finding specific apparent patterns. In classifying time-domain signals by specific apparent patterns, visibility graphs, or vertical visibility graphs (Lacasa et al. 2007), which convert a time series into an associated graph linking every bar with all those that can be seen from the top of a given bar, is one candidate tool that can be used in this research problem. On the other hand, as a tool in the family of visibility graphs, horizontal visibility algorithms (Luque et al. 2009), which finds links in only the horizontal direction from the top of a considered bar, is another candidate tool. In one application, Zhu et al. (2014) applied a horizontal visibility algorithm in an EEG problem. However, EMG signals generally have a specific pattern of isolated epochs with extremely high peaks. This specific pattern might not be applicable to horizontal visibility algorithms, as shown in some samples in the third column of Fig. 3. The obtained associated graphs shown under the horizontal visibility graphs for normal, ALS, and myopathic cases in the 3rd column of Fig. 3 reveal classification difficulty, while those of the vertical visibility algorithm shown in the 4th column markedly differentiate patterns of normal, ALS, and myopathy. These findings indicate that horizontal visibility algorithms cannot address this problem. Unlike horizontal visibility algorithms, vertical visibility algorithms are possible for differentiating signals, especially ALS and myopathy signals, since the differences in the vertical direction are comparatively more obvious due to the outstanding differences in signal peak and duration. Clearly, relations or links of all pulses with other pulses in vertical visibility in an epoch reveal distinct patterns among normal, ALS, and myopathy signals, as shown by examples in the fourth column of Fig. 3. Based on the vertical-visibility results of normal, ALS, and myopathy epochs shown in the 2nd, 4th, and 6th rows, respectively, the number of links and their relations in all pulses of each epoch clearly distinguish the disease identities. However, the number of nodes and their links may not be sufficient to classify disease types in some cases, such as in the example shown in Fig. 4. These two epochs (A and B) shown in the first column are clearly different to the naked eye. Their vertical-visibility features shown in the second column, however, look the same (Fig. 4d, h), which subsequently leads to a critical error. In fact, the 3rd and 5th pulses of the epochs are physically different, although the vertical visibility algorithm is not able to reflect this difference. Another feature implicitly used by humans for differentiating these cases is the differences in pulse length related to the peak of epoch as shown by a_i_ and b_i_ in Fig. 4c, g. Therefore, a peak in each epoch needs to be normalized in the same standard such as length “1” for a fair comparison. Apparently, the initial differences among all pulses in an epoch are other key features for signal-type differentiation, as shown in the third column (Fig. 4c, g). Therefore, these pulse length differences are proposed as weights in this study for extraction as another distinct feature. The differences between pulses in epoch A (a1, a2, a3, …) and those of epoch B (b1, b2, b3, …) are collected as weights in the form of an adjacency matrix, as shown in Fig. 5. The order of nodes (1, 2, 3, …, N) and their linked nodes (1, 2, 3, …, N) in the epoch are arranged in the matrix column and row directions, respectively. All elements in the matrix express the number of links. As a result, the adjacency matrix shown in Fig. 5a, representing the number of links obtained from the vertical visibility of epochs A and B, become exactly the same. The adjacency matrices in Fig. 5b, c representing the weights of all nodes and links in epochs of A and B, respectively, clearly show evident features.

## Proposed method of EMG-based feature extraction

Based on the aforementioned basic concept, our proposed method of EMG-based feature extraction for ALS and myopathy detection begins with preprocessing, followed by feature extraction and classification processes. Process overview of the proposed method is explained in “Overview processes of proposed method” section, and all proposed processes, including preprocessing, feature extraction, and classification, are described in “Preprocessing”–“Classification” sections, respectively.

### Overview processes of proposed method

**Fig. 6:**
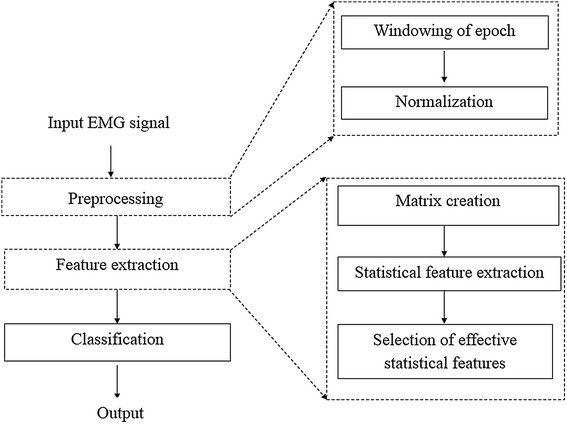
Proposed methods for EMG classification

A typical EMG-based classification system consists of three processes: preprocessing, feature extraction, and classification, as shown in Fig. 6. The first process of preprocessing is furthermore divided into (1) epoch windowing and (2) normalization. In these steps, epochs of recorded EMG signals are first detected, and the detected epochs are then normalized to a standard form wherein all peaks are adjusted to 1. In the next step of our proposed feature extraction process shown by the dotted box, normalized epochs are converted into features of vertical visibility where links and weights are measured, and matrices representing links and weights are subsequently formed. Statistical mechanics are used to evaluate the results of this feature-extraction process, and features, which are confirmed to be powerful for classification, are finally selected for the next classification process. The last step of classification use selected features as inputs to various classifiers, such as *k*-NN, MLPNN, and SVM classifiers, for detection of ALS and myopathy.

### Preprocessing

**Fig. 7:**
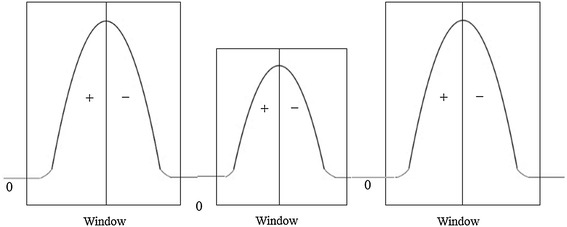
A synthesized signal for illustrating the widowing step in the preprocessing process

**Fig. 8:**
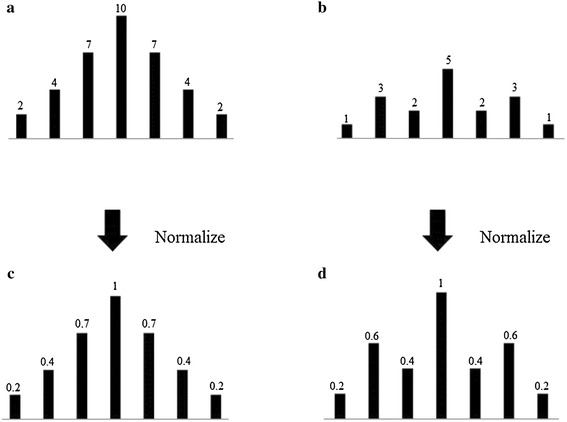
Normalization of signals. **a** Original pulses of epoch I, **b** original pulses of epoch II, **c** normalized pulses of epoch I, and **d** normalized pulses of epoch II

In the preprocessing process, EMG signals from normal, ALS, and myopathy cases are detected for epochs. As shown in Fig. 7, epochs are generally isolated among non-signal states such that the epoch boundaries are generally the borders between epochs and non-signal states. The curve of an epoch, which is scanned from left to right, theoretically starts with a positive slope and is followed by a negative slope, and the non-signal states located between the epochs are regarded as zero slope. Based on the pattern of the slopes, borders between an epoch and the surrounding non-signal states are detected, which becomes a window, and the peak pulse in the window is then considered as the center point of the epoch. The detected epoch peak is subsequently assumed to have an amplitude of “1”, and other pulse lengths in the epoch window are normalized to the peak, as shown in the epoch examples in Fig. 8. Epochs therefore are normalized via the same method.

### Feature extraction

In the feature extraction process, as shown by the second dashed rectangle in the flowchart in Fig. 6, a normalized epoch is first sampled based on the sampling theory, and the sampled pulses are then extracted for vertical visibility features including the number of node links and weights using the normalized weight vertical visibility algorithm (NWVVA) in the process of matrix creation. Those links and weights are put into matrix form, the feature matrices and obtained features are filtered and considered by statistical machines for selected powerful features in the next step of statistical feature extraction. Effective statistical features are selected during the step of the last process. The following section is divided into two-subsections, extraction of candidate features and feature finalization.

#### Matrix creation

**Fig. 9:**
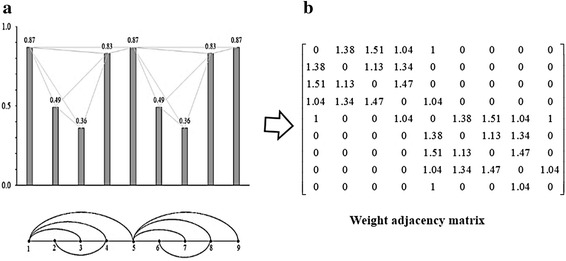
Illustration of the weight vertical visibility algorithm; **a** amplitudes and relation graph and **b** weight adjacency matrix for the amplitudes and relation graph in **a**

As shown by the example in Fig. 9, the pulses obtained based on the sampling theory are subjected to the vertical visibility algorithm, and the links of all nodes are counted, as shown in the bottom row of Fig. 9a. Simultaneously, the differences for all pulses compared with the linked nodes are measured according to their weights as shown in the upper row of Fig. 9a, and all weights are formed in an adjacency matrix as shown in Fig. 9b.

In the example shown in Fig. 9, nine sampling points are obtained. For a given sampling point or node, all other surrounding sampling peaks to which straight lines from the considered point can be drawn without any obstacles are defined as related to the sampling point, and these related nodes are counted and used to create an adjacency matrix. As show the sample of Fig. 9a, the sampling point 4 is related with sampling points 1 and 5 via high sight (the node is looking up) and 2 and 3 via low sight (the node is looking down). However, the sampling point 6–9 are hidden by sampling point 5, and therefore, that no relation is counted from them. To account for both the relation link and amplitude features, element W_*ij*_ of the weighted adjacency matrix is obtained as follows. If there exists a link between node i and j, W_*ij*_ is first set to 1 to account for the relation link, and then the absolute difference of the normalized amplitudes between nodes i and j is added, which produces element (W_*ij*_) of the matrix. If node i and j have no link, W_*ij*_ is set to zero. All diagonal elements (W_*ij*_), which indicate links with itself are set to zero.

The procedure of the aforementioned concept can be described based on normalized weight vertical visibility algorithm (NWVVA) as follows:

The Eq. (1) summarizes the calculation of W_*ij*_,

1 $$\text{W}_{ij}=\left\{ {\begin{array}{lll} {abs(x_i-x_j) + 1;} & {\text{where } {x_n}<{x_j}+(x_j-x_i)\frac{n-j}{j-i}, i<n<j,}\, \text{or}&{abs(i-j)=1} \\ 0; &\quad\quad{\text{elsewhere}}\end{array}}\right.$$

In the example shown in Fig. 9b, the values of the relations of the weighted adjacency matrix are determined as follows. There exists a link between the 1st and 2nd data points whose amplitudes are 0.87 and 0.49, respectively. Hence, W_*12*_ is the absolute value of (0.87–0.49); adding “1” equals 1.38, while the same procedure is applied to obtain other elements of the matrix. As a result, a weight adjacency matrix for this signal is obtained as shown in Fig. 9b.

#### Statistical feature extraction

In statistical feature extraction, it is complicated and redundant to classify epochs by some classifiers using perceptron data and the features extracted in the previous process. Because these features hold statistical characteristics in each target classified groups (normal, ALS, and myopathy), it is better for users to utilize statistical mechanics and statistical measurements as inputs to the appropriate classification tools. However, because not all statistical mechanics and measurements are effective for classification, a process of selecting effective statistical mechanics is needed in the learning state, which could be done in advance. Such a way to select effective statistical mechanics and measurements is introduced as a guideline as follows.

In the learning state, users should first calculate candidate features of the number of links and weights obtained via vertical visibility by using possible statistical mechanics and measurements and then consider selecting only the effective features based on the selected set of training signals. The selected statistical mechanics and measurements are then used to find final features in the testing state.

The statistical mechanics and measurements as candidates for selection in the learning state are introduced in “Average degree”–“Kurtosis” sections, respectively, as follows.

##### Average degree

An average degree (*AD*) indicates the average number of links that connect to one node in a network. The *AD* of a node in a graph is defined as (Barabasi 2012):

2 $$ AD = \frac{2L}{N}, $$

where *N* is the size of the network, and *L* is the number of links, which represents the total number of interactions between nodes and is given by

3 $$ L = \frac{1}{2}\mathop \sum \limits_{i = 1}^{N} k_{i} , $$

where *k*_*i*_ is the degree of the *i*th node in the network.

In the sample in Fig. 9, *L* = 16, and *N* = 9, hence *AD* = 3.55.

##### Average clustering coefficient

The average clustering coefficient (*ACC*) represents the relationship between the nodes in a complex network and describes the degree of clustering in the entire network. Let *C*_*i*_ be the local cluster coefficients of node i. Then, the ACC is the average of *C*_*i*_ over all nodes *i* = 1, …, *N* (Barabasi 2012):

4 $$ ACC = \frac{1}{N}\mathop \sum \limits_{i = 1}^{N} C_{i} , $$

where

5 $$ C_{i} = \frac{{2L_{i} }}{{k_{i} \left( {k_{i} - 1} \right)}}, $$

where *L*_*i*_ described by Eq. (3) is the number of links between the *k*_*i*_ neighbors of node *i*.

In the sample in Fig. 9, *N* = 9, *L*_*i*_: 4, 3, 3, 4, 6, 3, 3, 4, 2. As a result, *Ci* = 0.82, 1.29, 1.32, 0.82, 0.39, 1.29, 1.32, 0.82, and 1.02 for *i* = 1, 2, …,9, respectively; hence *ACC* = 1.01.

##### Transitivity

Transitivity (*T*) is defined as (Newman 2003):

6 $$ T = \frac{{3 \times {\text{number}}\;{\text{of}}\;{\text{triangles}}\;{\text{in}}\;{\text{network}}}}{{{\text{number}}\;{\text{of}}\;{\text{connected}}\;{\text{triples}}\;{\text{of}}\;{\text{vertices}}}}, $$

where a triangle is a set of tree vertices that are connected to one another, and a “connected triple” is a single vertex with adjacent edges connected to an unordered pair of vertices.

In the sample in Fig. 9, number of triangles in the network and connected triples are 74.73 and 92, respectively; hence *T* = 0.81.

##### Assortativity

Assortativity (*As*) is a correlation coefficient between the degrees of all nodes on two opposite ends of a link. It is defined as (Newman 2010):

7 $$ As = \frac{{S_{1} S_{e} - S_{2}^{2} }}{{S_{1} S_{3} - S_{2}^{2} }} $$

where $$ S_{e} = \sum\nolimits_{ij} {W_{ij} k_{i} k_{j} } $$, $$ S_{1} = \sum\nolimits_{i} {k_{i} } $$, $$ S_{2} = \sum\nolimits_{i} {k_{i}^{2} } $$, and $$ {\text{S}}_{3} = \sum\nolimits_{i} {k_{i}^{3} } $$.

A positive value of *As* indicates that the nodes tend to link to other nodes of an identical or similar degree. In the example in Fig. 9, *AS* = −0.32.

##### Density

Density (*Den*) represents the completeness of a group. The link density is defined as the proportion of the actual number of links to the maximum possible number of links among all nodes. *Den* is the ratio of the actual number of connections over the total number of possible connections (Newman 2010):

8 $$ Den = \frac{2L}{{N\left( {N - 1} \right)}} $$

In the example in Fig. 9, *L* = 16 and *N* = 9, hence Den = 0.44.

##### Central point dominance

Node betweenness centrality is the fraction of all the shortest paths in the network that contain a given node. Nodes with high values of betweenness centrality are part of many shortest paths. The betweenness centrality *B*_*u*_ of a vertex *u* is defined as follows (Costa et al. 2010):

9 $$ B_{u} = \mathop \sum \limits_{\text{ij}} \frac{{\upsigma\left( {i,u,j} \right)}}{{\upsigma\left( {i,j} \right)}} $$

in which σ (*i*, *u*, *j*) is the number of shortest paths between vertices *i* and *j* that pass through vertex *u*, σ (*i*, *j*) is the total number of shortest paths between i and j, and the sum is over all i,j pairs of distinct vertices. Central point dominance (*CPD*) is defined as follows (Costa et al. 2010):

10 $$ CPD = \frac{1}{N - 1}\mathop \sum \limits_{i = 1}^{N} \left( {B_{\hbox{max} } - B_{i} } \right) $$

where *B*_max_ represents the maximum betweenness in the network and *B*_*i*_ represents the node betweenness centrality.

In the example in Fig. 9, *B*_*u*_ = 10, 0, 0, 10, 34, 0, 0, 2, 0 and max(*B*_*u*_ = 34), hence *CPD* = 25.77.

##### Closeness centrality

Closeness (*CC*) is a measure of how long it takes to sequentially spread information from a node to all other nodes. In the classical definition of *CC*, the spread of information is modeled using the shortest paths (Newman 2010):

11 $$ CC_{i} = \frac{N}{{\mathop \sum \nolimits_{j} d_{ij} }} $$

In the example in Fig. 9, *d*_*ij*_ = $$ \left[ {\begin{array}{*{20}l} 0 \hfill & {0.72} \hfill & {0.66} \hfill & {0.96} \hfill & 1 \hfill & 0 \hfill & 0 \hfill & 0 \hfill & 0 \hfill \\ {0.72} \hfill & 0 \hfill & {0.88} \hfill & {0.74} \hfill & 0 \hfill & 0 \hfill & 0 \hfill & 0 \hfill & 0 \hfill \\ {0.66} \hfill & {0.88} \hfill & 0 \hfill & {0.68} \hfill & 0 \hfill & 0 \hfill & 0 \hfill & 0 \hfill & 0 \hfill \\ {0.96} \hfill & {0.74} \hfill & {0.68} \hfill & 0 \hfill & {0.96} \hfill & 0 \hfill & 0 \hfill & 0 \hfill & 0 \hfill \\ 1 \hfill & 0 \hfill & 0 \hfill & {0.96} \hfill & 0 \hfill & {0.72} \hfill & {0.66} \hfill & {0.96} \hfill & 1 \hfill \\ 0 \hfill & 0 \hfill & 0 \hfill & 0 \hfill & {0.72} \hfill & 0 \hfill & {0.88} \hfill & {0.74} \hfill & 0 \hfill \\ 0 \hfill & 0 \hfill & 0 \hfill & 0 \hfill & {0.66} \hfill & {0.88} \hfill & 0 \hfill & {0.68} \hfill & 0 \hfill \\ 0 \hfill & 0 \hfill & 0 \hfill & 0 \hfill & {0.96} \hfill & {0.74} \hfill & {0.68} \hfill & 0 \hfill & {0.96} \hfill \\ 0 \hfill & 0 \hfill & 0 \hfill & 0 \hfill & 1 \hfill & 0 \hfill & 0 \hfill & {0.96} \hfill & 0 \hfill \\ \end{array} } \right] $$ and *CC*_*i*_ = 0.07, 0.04, 0.04, 0.07, 0.08, 0.05, 0.05, 0.06, and 0.05, and hence *CPD* = 0.06.

##### Average shortest path (*ASP*)

A measure of the separation between two nodes in the graph is given by the *ASP* length, also known as the characteristic path length. It is defined as the mean of the lengths between all node pairs (Boccaletti et al. 2006):

12 $$ ASP = \frac{1}{{N\left( {N - 1} \right)}}\mathop \sum \limits_{i,j,i \ne j} d_{ij} $$

where *d*_*ij*_ is the length from node *i* to node *j*.

In the example in Fig. 9, *d*_*ij*_ = $$ \left[ {\begin{array}{*{20}l} 0 \hfill & {0.72} \hfill & {0.66} \hfill & {0.96} \hfill & 1 \hfill & 0 \hfill & 0 \hfill & 0 \hfill & 0 \hfill \\ {0.72} \hfill & 0 \hfill & {0.88} \hfill & {0.74} \hfill & 0 \hfill & 0 \hfill & 0 \hfill & 0 \hfill & 0 \hfill \\ {0.66} \hfill & {0.88} \hfill & 0 \hfill & {0.68} \hfill & 0 \hfill & 0 \hfill & 0 \hfill & 0 \hfill & 0 \hfill \\ {0.96} \hfill & {0.74} \hfill & {0.68} \hfill & 0 \hfill & {0.96} \hfill & 0 \hfill & 0 \hfill & 0 \hfill & 0 \hfill \\ 1 \hfill & 0 \hfill & 0 \hfill & {0.96} \hfill & 0 \hfill & {0.72} \hfill & {0.66} \hfill & {0.96} \hfill & 1 \hfill \\ 0 \hfill & 0 \hfill & 0 \hfill & 0 \hfill & {0.72} \hfill & 0 \hfill & {0.88} \hfill & {0.74} \hfill & 0 \hfill \\ 0 \hfill & 0 \hfill & 0 \hfill & 0 \hfill & {0.66} \hfill & {0.88} \hfill & 0 \hfill & {0.68} \hfill & 0 \hfill \\ 0 \hfill & 0 \hfill & 0 \hfill & 0 \hfill & {0.96} \hfill & {0.74} \hfill & {0.68} \hfill & 0 \hfill & {0.96} \hfill \\ 0 \hfill & 0 \hfill & 0 \hfill & 0 \hfill & 1 \hfill & 0 \hfill & 0 \hfill & {0.96} \hfill & 0 \hfill \\ \end{array} } \right] $$ and *N* = 9, and hence *ASP* = 0.37.

##### Global efficiency (*E*)

*E* is the average of the inverse shortest path length and is inversely related to the characteristic path length. The node eccentricity is the maximum shortest path length between a node and any other node (Boccaletti et al. 2006):

13 $$ {\text{E}} = \frac{1}{{N\left( {N - 1} \right)}}\mathop \sum \limits_{i,j,i \ne j} \frac{1}{{d_{ij} }} $$

In the example in Fig. 9, *d*_*ij*_ = $$ \left[ {\begin{array}{*{20}l} 0 \hfill & {0.72} \hfill & {0.66} \hfill & {0.96} \hfill & 1 \hfill & 0 \hfill & 0 \hfill & 0 \hfill & 0 \hfill \\ {0.72} \hfill & 0 \hfill & {0.88} \hfill & {0.74} \hfill & 0 \hfill & 0 \hfill & 0 \hfill & 0 \hfill & 0 \hfill \\ {0.66} \hfill & {0.88} \hfill & 0 \hfill & {0.68} \hfill & 0 \hfill & 0 \hfill & 0 \hfill & 0 \hfill & 0 \hfill \\ {0.96} \hfill & {0.74} \hfill & {0.68} \hfill & 0 \hfill & {0.96} \hfill & 0 \hfill & 0 \hfill & 0 \hfill & 0 \hfill \\ 1 \hfill & 0 \hfill & 0 \hfill & {0.96} \hfill & 0 \hfill & {0.72} \hfill & {0.66} \hfill & {0.96} \hfill & 1 \hfill \\ 0 \hfill & 0 \hfill & 0 \hfill & 0 \hfill & {0.72} \hfill & 0 \hfill & {0.88} \hfill & {0.74} \hfill & 0 \hfill \\ 0 \hfill & 0 \hfill & 0 \hfill & 0 \hfill & {0.66} \hfill & {0.88} \hfill & 0 \hfill & {0.68} \hfill & 0 \hfill \\ 0 \hfill & 0 \hfill & 0 \hfill & 0 \hfill & {0.96} \hfill & {0.74} \hfill & {0.68} \hfill & 0 \hfill & {0.96} \hfill \\ 0 \hfill & 0 \hfill & 0 \hfill & 0 \hfill & 1 \hfill & 0 \hfill & 0 \hfill & {0.96} \hfill & 0 \hfill \\ \end{array} } \right] $$ and *N* = 9, hence *E* = 0.54

##### Network diameter (*D*)

The diameter of a network, denoted by *D*, is the maximum shortest path in the network. It is the largest recorded distance between any node pairs (Boccaletti et al. 2006):

14 $$ D = \hbox{max} \left( {d_{ij} } \right) $$

In the example in Fig. 9, max(*d*_*ij*_) = 4, and hence *D* = 0.96.

##### Average weight

The average weight (*AW*) or strength of the network on the possible visible link is defined using the weight adjacency matrix as follows (Zhu et al. 2014):

15 $$ AW = \frac{1}{N}\mathop \sum \limits_{i} \mathop \sum \limits_{j} W_{ij} . $$

In the example in Fig. 9, $\mathop \sum \limits_{i} \mathop \sum \limits_{j} W_{ij} = 39.64$, and hence *AW* = 4.40.

##### Skewness

The skewness is the third standardized moment, defined as (Zhu et al. 2014):

16 $$ skewness = \frac{{\mathop \sum \nolimits_{i} \mathop \sum \nolimits_{j} \left( {W_{ij} - \mu } \right)^{3} /N}}{{s^{3} }} $$

where *μ* is the mean, and *s* is the standard deviation.

In the example in Fig. 9, *μ* = 0.49 and *s* = 0.62, and hence *skewness* = 0.56.

##### Kurtosis

The kurtosis is the fourth standardized moment, defined as (Zhu et al. 2014):

17 $$ kutosis = \frac{{\mathop \sum \nolimits_{i} \mathop \sum \nolimits_{j} \left( {W_{ij} - \mu } \right)^{4} /N}}{{s^{4} }} $$

where *μ* is the mean, and *s* is the standard deviation.

In the example in Fig. 9, *μ* = 0.49 and *s* = 0.62, and hence *kurtosis* = 1.64.

These calculation results obtained by the selected statistical mechanics explained above are evaluated by ANOVA (Wassernman 2013), and the evaluated results are used to construct feature vectors. These vectors would be classified into healthy, myopathy and ALS statuses, which is explained in the next section.

#### Selection of effective statistical features

**Fig. 10:**
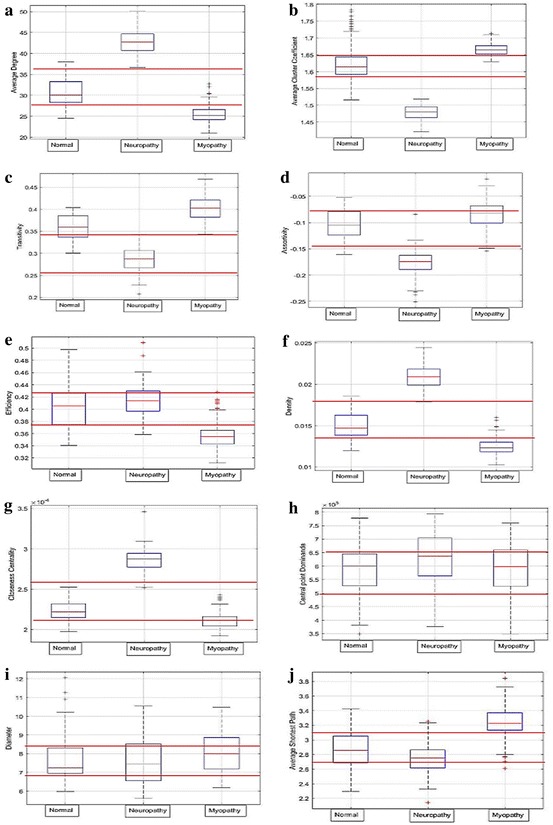
Distribution of network measurements for EMG signals; **a** average degree, **b** average cluster coefficient, **c** transitivity, **d** assortativity, **e** closeness centrality, **f** central point dominance, **g** density, **h** average shortest path, **i** network diameter, and **j** global efficiency

**Fig. 11:**
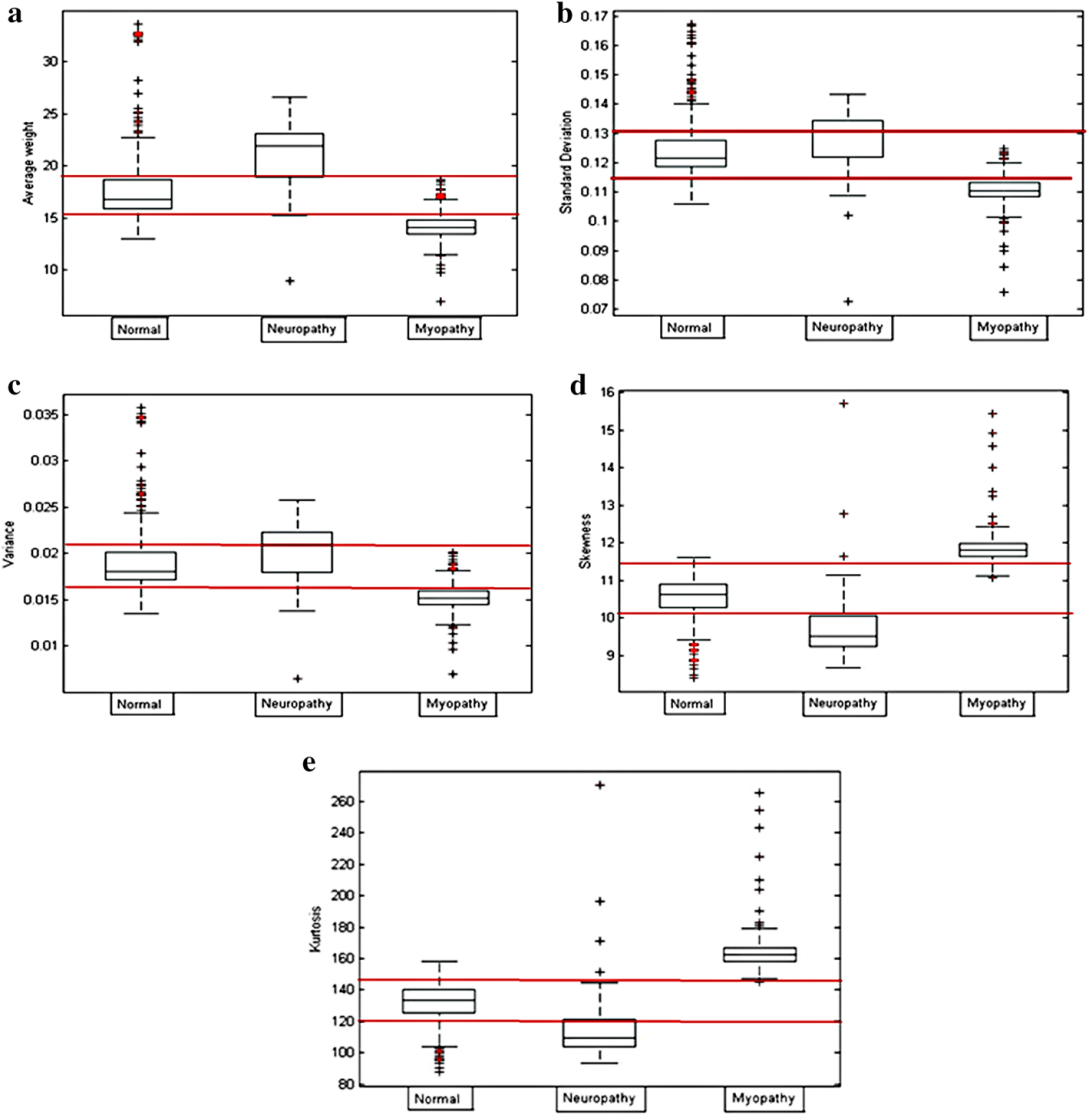
Distribution of statistics of the weight adjacency matrix: **a** average weight, **b** standard deviation, **c** variance, **d** skewness, and **e** kurtosis

In the learning state, users must initially perform pre-testing on some known samples to determine effective statistical mechanics and measurements for the testing state. During pre-testing, users should pick existing statistical mechanics and measurements, as described in the previous subsection, and perform calculations with the known samples as training samples after forming their weight adjacency matrices. Based on the calculation results of the training samples, users should select only mechanics and measurements, which can clearly classify normal, ALS, and myopathy without any overlap, as effective tools for the testing state. Some tools such as ANOVA (Wassernman 2013) that can calculate independence levels among the training samples of the three groups (normal, ALS, and myopathy) can be theoretically used to finalize statistical mechanics and measurements, which are effective in the testing state. In an example of the statistical mechanics shown in Fig. 10, the training results obtained by the average degree in Fig. 10a, the average cluster coefficient in Fig. 10b, and the density in Fig. 10f, by which those three groups are perfectly separate, are selected as effective statistical mechanics. In another example of statistical measurements shown in Fig. 11, the average weight in Fig. 11a, the skewness in Fig. 11d, and the kurtosis in Fig. 11e, which clearly classify the three groups, are selected as effective statistical measurements for use in the testing state.

In the aforementioned examples, average degree, average cluster coefficient, density, average weight, skewness, and kurtosis are statistically selected as six effective features. However, users are recommended to undertake this type of pre-testing or training using their own samples to obtain effective features for their datasets.

### Classification

**Fig. 12:**
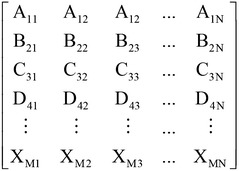
Feature vector matrix

In classification, the finalized features are converted into vector form, as shown in Fig. 12. The vector has M × N dimensions, where M and N represent the effective features and number of test datasets, respectively. The vectors are fed to the classifiers to classify healthy, myopathy and ALS statuses. Users are advised to choose a classifier or classifiers that work for their applications. In this paper, a *k*-nearest neighbor classifier (Cover and Hart 1967), a multilayer perceptron neural network (Haykin 1994), and a support vector machine (Krebel 1999) are recommended tools for classification.

## Datasets and experimental results

### Datasets

In our experiments, the databases 1 (Physionet 2016) and 2 (Nikolic 2001) used in the conventional methods are employed under the objective of fair comparison with the results of conventional methods, and the results classified by the *k*-NN, MLPNN, and SVM classifiers are shown and compared with those in the conventional methods, as follows.

**Fig. 13:**
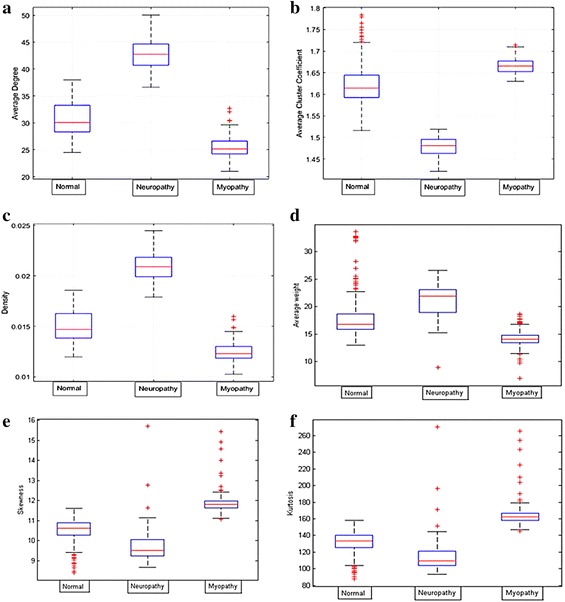
Distribution of database 1 for three classes of neuromuscular disorders: **a** average degree, **b** average cluster coefficient, **c** density, **d** average weight, **e** skewness, and **f** kurtosis

For both databases, each dataset in the time series was transformed using the weight vertical visibility algorithm (NWVVA), and the weight adjacency matrix was obtained. The network measurements including the average degree, average clustering coefficient, density, average weight, skewness, and kurtosis were calculated. For database 1, the distribution of these measurements for each dataset was plotted and is illustrated in Fig. 13. These measurements were employed to generate a feature vector for classification.

**Fig. 14:**
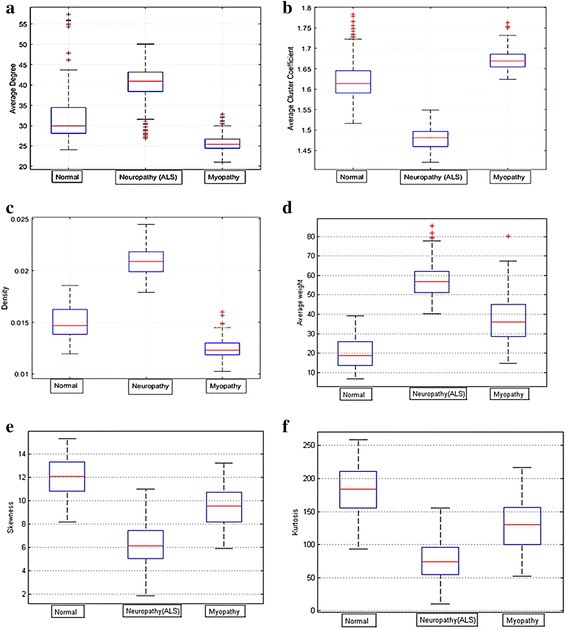
Distribution of database 2 based on three classes of neuromuscular disorders: **a** average degree, **b** average cluster coefficient, **c** density, **d** average weight, **e** skewness, and **f** kurtosis

For database 2, the network measurements for each group were plotted and compared to analyze the distribution, as shown in Fig. 14. From each measurement on both databases as shown in Figs. 13 and 14, the similarities were in the same trend.

We used ANOVA to verify whether the different values obtained for the three different groups represent significant differences. The p values for both databases as shown in Table 1 are very close to zero and therefore indicate that the differences are significant.

**Table 1:** The *p* values of the statistical mechanics for databases 1 and 2 using ANOVA

Feature extraction	*p* value	
	Database 1	Database 2
AD	*p* < 0.001	*p* < 0.001
ACC	*p* < 0.001	*p* < 0.001
Den	*p* < 0.001	*p* < 0.001
AW	*p* < 0.001	*p* < 0.001
Skewness	*p* < 0.001	*p* < 0.001
Kurtosis	*p* < 0.001	*p* < 0.001

### Experimental results

We classified EMG signals into three categories: normal, myopathy, and neuropathy. We divided a dataset into training and testing data with ratios of testing data of 20, 40, 50, 60, and 80%. Additionally, we employed a fivefold cross-validation criterion to the training and testing data. The performance of the classifier was evaluated based on calculating of the following statistical parameters:

- *Specificity* the number of correctly classified normal subjects divided by the number of total normal subjects.
- *Sensitivity (myopathy)* the number of correctly classified subjects suffering from myopathy divided by the number of total subjects suffering from myopathy.
- *Sensitivity (neuropathy)* the number of correctly classified subjects suffering from neuropathy divided by the number of total subjects suffering from neuropathy.
- *Total classification accuracy* the number of correctly classified subjects divided by the number of total subjects.

The performance of the classifiers was evaluated by computing the statistical parameters, as shown in Table 2 for databases 1 and 2, respectively.

**Table 2:** Summary of the classification performance of the proposed method

Test (%)	Statistical parameter	Database 1			Database 2		
		*k*-NN	MLPNN	SVM	*k*-NN	MLPNN	SVM
20	Specificity	94.86 ± 1.48	96.08 ± 1.24	98.68 ± 1.02	94.50 ± 1.18	95.37 ± 1.64	97.86 ± 0.84
	Sensitivity (neuropathy)	96.46 ± 0.68	97.18 ± 0.96	98.98 ± 0.58	95.20 ± 1.69	96.26 ± 1.86	98.26 ± 0.94
	Sensitivity (myopathy)	98.28 ± 1.27	98.82 ± 1.20	99.86 ± 0.86	97.90 ± 1.20	97.23 ± 1.02	98.98 ± 0.62
	Total classification accuracy	*96.53 ± 0.92*	*97.36 ± 0.74*	*99.17 ± 0.68*	*95.87 ± 0.85*	*96.28 ± 0.62*	*98.36 ± 0.48*
40	Specificity	92.54 ± 1.84	95.23 ± 1.84	98.42 ± 1.62	91.95 ± 2.01	94.78 ± 1.82	97.08 ± 1.58
	Sensitivity (neuropathy)	94.98 ± 1.92	96.02 ± 1.69	98.21 ± 1.82	94.55 ± 1.57	95.72 ± 2.02	98.26 ± 1.42
	Sensitivity (myopathy)	98.18 ± 0.74	98.18 ± 0.92	99.26 ± 0.94	98.15 ± 0.91	97.56 ± 1.24	98.86 ± 1.08
	Total classification accuracy	*95.23 ± 0.86*	*96.47 ± 0.76*	*98.63 ± 0.82*	*94.88 ± 0.78*	*96.02 ± 0.89*	*98.06 ± 0.68*
50	Specificity	92.46 ± 2.26	95.46 ± 1.92	97.04 ± 2.12	91.24 ± 2.58	94.18 ± 2.26	96.12 ± 1.98
	Sensitivity (neuropathy)	93.78 ± 1.69	96.28 ± 1.96	98.98 ± 1.65	92.68 ± 1.72	95.23 ± 1.68	98.62 ± 1.28
	Sensitivity (myopathy)	98.75 ± 0.94	98.92 ± 0.81	99.98 ± 1.46	98.04 ± 0.83	97.98 ± 1.24	99.24 ± 0.98
	Total classification accuracy	*94.99 ± 0.83*	*96.88 ± 0.68*	*98.63 ± 0.94*	*93.99 ± 0.97*	*95.79 ± 0.72*	*97.99 ± 0.82*
60	Specificity	91.89 ± 2.34	93.49 ± 3.02	96.78 ± 2.98	90.97 ± 2.00	92.43 ± 2.98	96.02 ± 2.46
	Sensitivity (neuropathy)	91.94 ± 2.28	95.82 ± 2.46	97.69 ± 2.46	91.10 ± 2.43	94.39 ± 2.04	96.92 ± 1.48
	Sensitivity (myopathy)	97.64 ± 0.87	97.46 ± 1.04	99.43 ± 1.24	96.73 ± 0.64	95.45 ± 1.12	98.42 ± 1.12
	Total classification accuracy	*93.82 ± 0.93*	*95.59 ± 0.68*	*97.96 ± 0.86*	*92.93 ± 0.75*	*94.09 ± 0.74*	*97.12 ± 0.84*
80	Specificity	88.46 ± 2.46	92.63 ± 1.92	94.98 ± 1.95	87.73 ± 2.05	91.94 ± 2.02	94.76 ± 3.98
	Sensitivity (neuropathy)	88.12 ± 1.24	93.82 ± 1.28	97.84 ± 1.74	87.65 ± 1.61	92.83 ± 1.76	97.14 ± 1.92
	Sensitivity (myopathy)	98.06 ± 1.29	95.92 ± 1.24	98.68 ± 1.84	97.60 ± 1.09	93.92 ± 1.82	97.64 ± 2.04
	Total classification accuracy	*91.54 ± 0.83*	*94.12 ± 0.98*	*97.16 ± 0.95*	*90.99 ± 0.82*	*92.89 ± 0.94*	*96.51 ± 0.96*

Italic values mean total classification accuracy

All values show in mean ± standard deviation

We report the performance attained by the SVM classifier compared with previous works that employed different methods, as specified in Table 3. The total classification accuracies of the proposed method are outstanding for both databases.

**Table 3:** Summary of the classification performances of previous methods

Method (feature + classification)	Total classification accuracy (%)
Database 1	
RQA + SVM (Sultornsanee et al. 2011)	98.28
VVA + SVM (Artameyanant et al. 2014)	99.07
WVA + MLPNN (Artameyanant et al. 2015)	94.73
Proposed method	99.17
Database 2	
AR + WNN (Subasi et al. 2006)	90.70
CWT + SVM (Istenic et al. 2010)	70.40
AR + neuro-fuzzy system (Kocer 2010)	90.00
AR-DWT + DFNN (Subasi 2012a, b)	94.00
DWT + ESVM (Subasi 2013a)	97.00
DWT + PSO-SVM (Subasi 2013b)	97.41
DWT + random forest (Gokgoz and Subasi 2015)	96.67
Proposed method	98.36

## Discussion

This paper proposes a method of EMG-based feature extraction using a normalized weight vertical visibility algorithm for ALS and myopathy detection. Due to the effectiveness of specific features of the vertical visibility algorithm with normalized weights, which are well matched with the patterns of ALS and myopathy signals, the proposed method yields better classification accuracy results compared with conventional methods as shown in Table 3. For studies targeting applications in medicine, which is critical for improving human life, the experimental results should ideally be perfect without any errors. However, the proposed method contributes to a new approach, which currently corresponded to best accuracy results that approached 100%. Research on this topic should be accepted and continue to be studied until the results successfully meet the final goal. Regarding errors in the experiments, their causes and how to prevent errors are analyzed and discussed as follows.

**Fig. 15:**
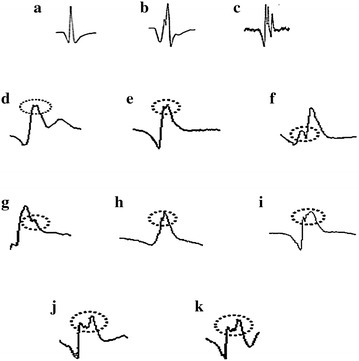
Errors in normal, ALS, and myopathy detection. **a**–**c** Typical patterns of normal, ALS, and myopathy, **d**–**f** normal detected as ALS, **g**–**i** ALS detected as normal, and **j**, **k** myopathy detected as ALS

Unlike typical signal patterns representing normal, ALS, and myopathy statuses, as shown in Fig. 15a–c, respectively, the errors that occurred can be grouped based on their causes into three cases: normal cases categorized as ALS, as shown in Fig. 15d–f; ALS cases categorized as normal, as shown in Fig. 15g–i; and myopathy cases categorized as ALS, as shown in Fig. 15j, k. For the first type of error corresponding to normal cases categorized as ALS, as shown in Fig. 15d–f, various sources of noise are regarded to have affected the signal and to have caused a transitional phenomenon denoted by the dashed circle that the classifier identifies as features of ALS. As a solution in this case, smoothing tools and low-pass filters should be considered in the pre-processing step. For the second type of error corresponding to ALS cases categorized as normal, as shown in Fig. 15g–i, various sources of noise also affect the signal and decrease the distinct features of ALS, as indicated by the dashed circle. Some enhancement processes should be considered as a solution. For the last type of error corresponding to myopathy cases categorized as ALS, as shown in Fig. 15j, k, the key features of myopathy indicated by the dashed circle are damaged by noise such that the classifier misses the pattern matching. Some enhancements, such as high-pass filters, should be considered in the pre-processing step as a solution. Because the second and last types of error are negative faults, which must be critically addressed for user-safety, adding a combination of low-pass filtering for noise reduction and high-pass filtering for feature enhancement may be a viable approach in future research.

The proposed method of EMG-based feature extraction using a normalized weight vertical visibility algorithm for myopathy and ALS detection improves classification accuracy and advantages. To obtain improved accuracy, computational complexity and time implicitly become disadvantages as trade-offs. Although the increase in computational time is often considered in comparisons with conventional methods, the necessary computational time in the proposed method is on the order of milliseconds, which is practically acceptable due to prominent improvements in current computing technologies.

During the final classification step of the proposed method, some popular classifiers such as *k*-NN, MLPNN, and SVM classifiers, were recommended and tested here. Users are recommended to find their own appropriate tools, which should match their applications. As shown in Table 2, the *k*-NN, MLPNN, and SVM classifiers yielded excellent accuracies as approximately 96, 97, and 98%, respectively. Although the results show that the SVM classifier, which yielded the highest accuracy, should be recommended as the classification tool in terms of accuracy, the accuracy differences compared with the other classifiers were not extremely high. In some applications that require highly efficient training with low complexity, *k*-NN classifiers should be considered as another choice. On the other hand, MLPNN classifiers, which are theoretically designed as a tool to address complicated classification with slightly high complexity, could be a compromise in some applications that require some level of complexity.

## Conclusions

This paper proposes a method of EMG-based feature extraction using a normalized weight vertical visibility algorithm for myopathy and neuropathy detection. In the proposed method, EMG signals representing muscle responses were sampled based on the sampling theory for reversible discrete pulses, and the features of the obtained pulses were then extracted via a vertical visibility algorithm with their normalized weights. An adjacent matrix, whose elements represent links between nodes and their weights, was accordingly created and employed to extract statistical features using statistical mechanics and measurements. These statistical features were finally classified using *k*-NN, MLPNN, and SVM classifiers into normal, ALS, and myopathic cases. To evaluate the performance of the proposed method, experiments were performed on conventional 2 databases, and the results revealed 98.36% accuracy, which is approximately 2% improvement compared with conventional methods.

## Abbreviations

ALS: amyotrophic lateral sclerosis
EMG: electromyography
NWVVA: normalized weight vertical visibility algorithm
*k*-NN: *k*-nearest neighbor
MLPNN: multilayer perceptron neural network
SVM: support vector machine
FFT: fast Fourier transform
AR: autoregressive spectral models
FHVA: fast weighted horizontal visibility algorithm
*AD*: average degree
*ACC*: average cluster coefficient
*T*: transitivity
*As*: assortativity
*Den*: density
*CPD*: central point dominance
*CC*: closeness centrality
*ASP*: average shortest path
*E*: global efficiency
*D*: network diameter
*AW*: average weight
